# A brighter force gauge for cells

**DOI:** 10.7554/eLife.38959

**Published:** 2018-07-19

**Authors:** Victor Pui-Yan Ma, Khalid Salaita

**Affiliations:** 1 Department of ChemistryEmory UniversityAtlantaUnited States; 2 The Wallace H. Coulter Department of Biomedical EngineeringEmory UniversityAtlantaUnited States; 3 Winship Cancer InstituteEmory UniversityAtlantaUnited States

**Keywords:** mechanobiology, vinculin, tension sensor, focal adhesion, FRET, None

## Abstract

An improved biosensor sheds new light on tension within proteins.

**Related research article** LaCroix AS, Lynch AD, Berginski ME, Hoffman BD. 2018. Tunable molecular tension sensors reveal extension-based control of vinculin loading. *eLife* **7**:e33927. doi: 10.7554/eLife.33927

Under a microscope, cells seem static, but in reality they are constantly pulling and pushing with miniscule forces. Despite being very small (about one millionth the weight of a grain of rice), these forces are important for a number of processes, including wound healing and the immune response. They can also regulate the cell’s fate during development (Mammoto et al., 2013).

To better understand the crosstalk between force and biochemistry in cells, the research community has developed smaller and smaller probes to measure forces (Helenius et al., 2008; Liu et al., 2017). A major breakthrough, reported just under a decade ago, was the development of a genetically encoded force gauge (Grashoff et al., 2010). This biosensor worked like a macroscopic tension gauge in that it contained a ‘spring’ (that stretched when pulled) and a ‘ruler’ (to measure how much the spring extended).

The spring element in the biosensor was adopted from a segment of spider silk and included a 40-amino acid polypeptide chain that formed a random coil. To measure how much it extended under force, fluorescent proteins were engineered at each end of the polypeptide. This pair of proteins was carefully chosen such that energy released after exciting one (the ‘donor’) with a light source was transferred to the other (the ‘acceptor’), causing it to emit light of a different wavelength. This phenomenon, named Förster resonance energy transfer (FRET), only occurs if the proteins are close enough, and it decreases when they move apart. As such, the FRET signal essentially represents the ruler that measures the length of the polypeptide coil.

This tension sensor module, or TSMod for short, was transformative and opened the door to mapping the forces experienced by a number of different mechanosensitive proteins, both in vitro and in vivo (Cost et al., 2015). Yet it was challenging to use, mostly because it lacked sensitivity (Eder et al., 2017). Part of the problem was that the FRET signal was weak, even when the proteins were close to each other. It was also made even weaker because it was concealed by the background glow from other parts of the cell that naturally fluoresce over similar wavelengths (e.g. mitochondria and lysosomes).

Now, in eLife, Andrew LaCroix, Andrew Lynch, Matthew Berginski and Brenton Hoffman of Duke University report how they completely re-engineered the probe to improve its performance (LaCroix et al., 2018). LaCroix et al. first systematically tested different pairs of fluorescent proteins, and whittled away the ‘linker’ region between the fluorescent proteins and the spring element (Austen et al., 2013). They also identified a ‘softer’ and less structured polypeptide spring (Evers et al., 2006), which further maximized the FRET signal (Figure 1).

**Figure 1.**
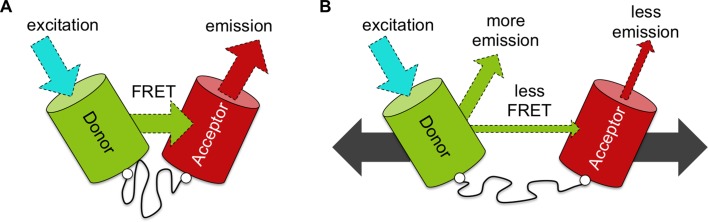
An improved biosensor to visualize tension in mechanosensitive proteins. (**A**) Like the original, the optimized tension sensor module contains a spring element (wavy line) attached to two fluorescent proteins (colored cylinders) via linker regions (white circles). However, while the original used cyan and yellow fluorescent proteins, the new version uses a green-red pair. Specifically, a green fluorescent protein called Clover acts as the ‘donor’ (green cylinder), and a red fluorescent protein called mRuby2 acts as the ‘acceptor’ (red cylinder). Excitation of the donor with cyan light causes it to give off a bright green light. If the donor is close enough to the acceptor – for example, because the spring is at rest – the fluorescence from the donor can be transferred to the acceptor via a process called FRET (see main text): the acceptor then emits red light. Dashed arrows of different colors indicate light of different wavelengths. (**B**) Via genetic engineering, this sensor module can be inserted within proteins of interest. If that protein is put under tension (gray solid arrows), the donor and acceptor proteins are pulled apart. This causes the amount of energy transferred by FRET to decrease, increasing emission of green light from the donor and reducing emission of red light from the acceptor. As such the ratio of emission at these two wavelengths provides a measure of how much the spring is extended, which gives an indication of the forces experienced within the protein of interest.

The optimized TSMod outperforms the original when tested in buffer. However, the gains in performance were lost when the new TSMod was tested in cells. This is an important warning to all researchers developing probes that are dedicated to measuring the forces acting on real cells but are calibrated away from real cells. Nonetheless, based on the data, LaCroix et al. developed a computational model that predicts how the spring element would behave inside cells. Using this model, they then identified the optimal peptide length to measure forces in vinculin, an important force-sensitive protein that is often used to evaluate the performance of this kind of biosensor (Schwartz, 2010).

With the aid of the computational modeling, the optimized TSMod mapped vinculin tension within cells much better than its predecessors. As a result of this improved performance, LaCroix et al. found a tension gradient across vinculin molecules within focal adhesions – the microstructures that anchor cells to their external environment. This asymmetry, which suggests that tension can be transmitted unevenly across the focal adhesion, was not detectable using with the original TSMod. Furthermore, using three optimized TSMods with springs of different lengths, LaCroix et al. showed that level of tension experienced by each sensor was different, but that all three sensors extended to approximately the same length. This result is intriguing because it suggests that focal adhesion formation and cell spreading may be governed by the physical extension of adaptor proteins (i.e., by how long they are), rather than the absolute magnitude of the forces they transmit (i.e. the level of tension they experience).

Another important result comes from systematic simulations that provide a ‘cheat-sheet’ to guide cell biologists in using the most optimal tension probe for a desired force range and range of light wavelengths. Those familiar with the original TSMod know that it often required extensive trial and error, and this road map will make the process more rational and predictable.

Moving forward, it remains to be seen whether other adaptor proteins within the focal adhesion complex, or other mechanosensitive molecules in general, get extended in the way that vinculin does. Nonetheless, the work by LaCroix et al. should motivate other researchers to look at other systems and processes that involve the transmission of forces (such as T cell receptors and the Notch-Delta signaling pathway; Liu et al., 2016; Luca et al., 2017). This finding could be the proverbial tip-of-the-iceberg for new discoveries in mechanobiology research.
